# RACIAL/ETHNIC DISPARITIES IN TRAJECTORIES OF HOSPITALIZATIONS AMONG PATIENTS WITH HEART FAILURE

**DOI:** 10.1093/geroni/igae098.3300

**Published:** 2024-12-31

**Authors:** Radha Dhingra, Hanzhang Xu, Bradley Hammill, Scott Lynch, Jessica West, Michael Green, Matthew Dupre

**Affiliations:** Duke University, Durham, North Carolina, United States; Duke University, Durham, North Carolina, United States; Duke University, Durham, North Carolina, United States; Duke University, Durham, North Carolina, United States; Duke University, Durham, North Carolina, United States; Duke University, Durham, North Carolina, United States; Duke University, Durham, North Carolina, United States

## Abstract

Little is known about racial/ethnic differences in the long-term patterns of hospitalization following the diagnosis of heart failure (HF). We examined racial/ethnic differences in trajectories of hospital admissions following a diagnosis of HF and assessed the factors contributing to the long-term risks of hospitalization. We included 5,606 patients with newly-diagnosed HF between January 2015 and July 2018 in the Duke University Health System and followed them for up to five years. Group-based trajectory models were used to identify trajectories of all-cause admissions following the diagnosis of HF. Multinomial logistic regression models were used to identify patient characteristics associated with the trajectories of admissions. Approximately 45.6% (N=2,556) of HF patients had low risks of hospitalization, 36.6% (N=2,052) had elevated risks of admission shortly after diagnosis (early risk), 9.9% (N=553) had elevated risks at later stages of illness (late risk), and 7.9% (N=445) had consistently high risks of hospitalization. Non-Hispanic Black patients were more likely to exhibit early risks of hospitalization (odds ratio, [95% CI]: OR=1.33, [1.16-1.52]; P<.001), late risks of hospitalization (OR=1.92, [1.58-2.34]; P<.001), or consistently high risks of hospitalization (OR=1.89, [1.52-2.35]; P<.001) compared with non-Hispanic White patients. Diabetes, chronic kidney disease, and residence in a disadvantaged neighborhood significantly contributed to the excess risks of admissions among non-Hispanic Black patients. Overall, these findings have important implications for identifying the social and clinical factors associated with racial disparities in the timing and frequency of hospital admissions in HF patients.

